# The Effect of Chronic Iloperidone Treatment on Cytochrome P450 Expression and Activity in the Rat Liver: Involvement of Neuroendocrine Mechanisms

**DOI:** 10.3390/ijms22168447

**Published:** 2021-08-06

**Authors:** Przemysław J. Danek, Wojciech Kuban, Władysława A. Daniel

**Affiliations:** Department of Pharmacokinetics and Drug Metabolism, Maj Institute of Pharmacology, Polish Academy of Sciences, Smętna 12, 31-343 Kraków, Poland; danek@if-pan.krakow.pl (P.J.D.); kuban@if-pan.krakow.pl (W.K.)

**Keywords:** iloperidone, prolonged administration, rat liver, cytochrome P450 expression/activity, hormone levels, neuroendocrine regulation

## Abstract

In order to achieve a desired therapeutic effect in schizophrenia patients and to maintain their mental wellbeing, pharmacological therapy needs to be continued for a long time, usually from the onset of symptoms and for the rest of the patients’ lives. The aim of our present research is to find out the in vivo effect of chronic treatment with atypical neuroleptic iloperidone on the expression and activity of cytochrome P450 (CYP) in rat liver. Male Wistar rats received a once-daily intraperitoneal injection of iloperidone (1 mg/kg) for a period of two weeks. Twenty-four hours after the last dose, livers were excised to study cytochrome P450 expression (mRNA and protein) and activity, pituitaries were isolated to determine growth hormone-releasing hormone (GHRH), and blood was collected for measuring serum concentrations of hormones and interleukin. The results showed a broad spectrum of changes in the expression and activity of liver CYP enzymes, which are important for drug metabolism (CYP1A, CYP2B, CYP2C, and CYP3A) and xenobiotic toxicity (CYP2E1). Iloperidone decreased the expression and activity of CYP1A2, CP2B1/2, CYP2C11, and CYP3A1/2 enzymes but increased that of CYP2E1. The CYP2C6 enzyme remained unchanged. At the same time, the level of GHRH, GH, and corticosterone decreased while that of T_3_ increased, with no changes in IL-2 and IL-6. The presented results indicate neuroendocrine regulation of the investigated CYP enzymes during chronic iloperidone treatment and suggest a possibility of pharmacokinetic/metabolic interactions produced by the neuroleptic during prolonged combined treatment with drugs that are substrates of iloperidone-affected CYP enzymes.

## 1. Introduction

Cytochrome P450 enzymes (CYPs) are a large class of heme-containing monooxygenases that catalyze NADPH-dependent C-, N-, and S-oxidation and O-, N-, and S-dealkylation of substrates. CYP enzymes are responsible for the metabolism of endogenous substrates, such as hormones, vitamins, arachidonic acid, bile acid, or steroids including neurosteroids, and for catalyzing the oxidation of exogenous substrates, such as xenobiotics and drugs of different pharmacological groups, including psychotropics [1,2].

Iloperidone is an atypical neuroleptic drug approved for the treatment of acute schizophrenia in adult patients [3]. Iloperidone, like other atypical neuroleptics, displays high and strong antagonistic activity and higher affinity for serotonin 5-HT_2A_ than dopamine D_2_ receptors [4]. It also has a high affinity for α_1_ and α_2_ adrenoreceptors and for dopamine D_3_ receptors; moderate affinity for dopamine D_4_, and serotonin 5-HT_6_ and 5-HT_7_; low affinity for serotonin 5-HT_1A_, dopamine D_1_, and histamine H_1_ receptors; and no affinity for cholinergic muscarinic receptors [5,6,7]. The drug is metabolized mainly by CYP2D6 (via carbonyl reduction and hydroxylation) and to a lesser degree by CYP3A4 (via O-demethylation) [3]. Compared with other typical neuroleptics, iloperidone is associated with a low incidence of akathisia and extrapyramidal symptoms [8] and a low propensity to elevate prolactin levels or to change metabolic parameters [9].

The physiological and pharmacological regulation of cytochrome P450 activity in the liver may proceed via different direct and indirect pathways. The direct mechanisms include drug binding to the enzyme, which can lead to the inhibition of enzyme activity via a competitive, noncompetitive, or mixed mechanism [10,11]. On the other hand, the indirect regulation of enzyme activity may involve both peripheral (e.g., drug binding to the hepatic membrane, and cytosolic or nuclear receptors) and central mechanisms. The central mechanisms engage the brain’s nervous and endocrine systems, thus leading to the neuroendocrine regulation of CYP gene expression. This kind of mechanism has been shown to involve the brain’s dopaminergic, noradrenergic, and serotonergic systems in combination with the hypothalamic endocrine center [12,13,14,15].

Considering the abovementioned possibilities of drug influence on cytochrome P450, in vitro models may be used to examine the direct and indirect peripheral mechanisms taking place in the liver (e.g., liver slices, hepatocyte cultures, liver microsomes, or cDNA-expressed CYPs). However, studying the drug effects on CYP regulation in the liver mediated by central mechanisms requires in vivo models, whereby the brain’s nervous system (via its receptors located on hypothalamic endocrine cells) can act on peripheral endocrine glands and, in turn, on liver function [15,16,17].

To produce and maintain the therapeutic effect in schizophrenia, neuroleptic drugs have to be administered to patients for a long period of time (months or years). It creates the possibility for different kinds of interactions (direct and indirect) of the applied drugs with receptors, enzymes, or genes encoding those biologically active proteins. Thus, drugs acting on the central nervous system should be investigated both in vitro and in vivo to discover all possibilities of their interaction with cytochrome P450. Our previous studies showed that iloperidone directly inhibited CYP2C19, CYP2D6, and CYP3A4 in liver microsomes via mixed, competitive, or noncompetitive mechanisms, respectively [18], and diminished the CYP3A4 expression in hepatocyte cultures [19]. The aim of our present research was to find out the in vivo effect of a two-week treatment with iloperidone on the expression and activity of liver cytochrome P450 enzymes, which are known to undergo neuroendocrine regulation.

## 2. Results

### 2.1. The Effect of Chronic Treatment with Iloperidone on the CYP Activity in Rat Liver Microsomes

The administration of iloperidone to rats for two weeks decreased the activity of CYP1A, i.e., C-8-hydroxylation and 3-N-demethylation, down to 68% and 71% of the control, respectively. Iloperidone diminished the activity of CYP2B (testosterone 16β-hydroxylation) down to 67% of the control; that of CYP2C11 (testosterone 2α- and 16α-hydroxylation) to 69% and 67% of the control, respectively; and that of CYP3A (testosterone 2β- and 6β-hydroxylation) to 83% and 71% of the control, respectively. In contrast, prolonged treatment of animals with iloperidone increased the activity of CYP2E1 (chlorzoxazone 6-hydroxylation) up to 115% of the control.

**Figure 1:**
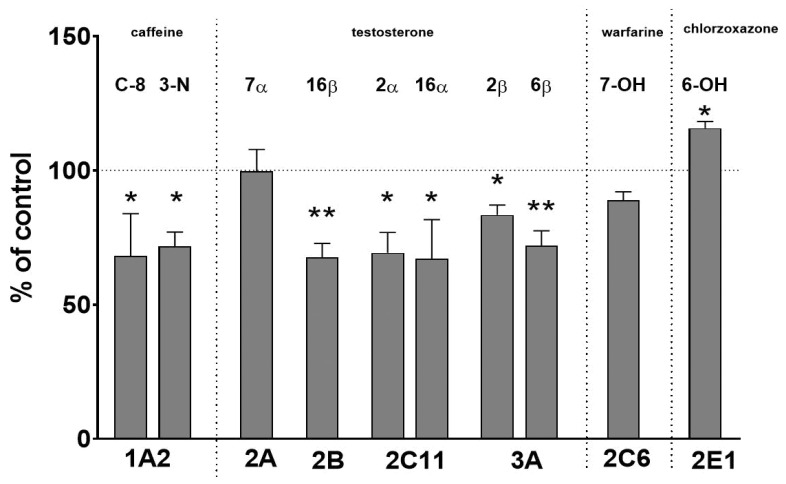
The effect of two-week treatment with iloperidone on cytochrome P450 enzyme activities, measured as the rates of CYP-specific reactions in rat liver microsomes: caffeine 8-hydroxylation and 3-N-demethylation (CYP1A); testosterone 7α- (CYP2A), 16β- (CYP2B), 2α-, and 16α- (CYP2C11); 2β- and 6β- (CYP3A) hydroxylation; warfarin 7-hydroxylation (CYP2C6); and chlorzoxazone 6-hydroxylation (CYP2E1). All values are shown as the mean ± S.E.M. (*n* = 12). Statistical significance was assessed by Student’s t-test and marked as * *p* < 0.05; ** *p* < 0.01, compared with the control. The control values (picomoles per milligram protein per minute) are as follows: 11.28 ± 3.07 (8-hydroxy-caffeine); 1.74 ± 0.6 (3-N-demethyl-caffeine); 134 ± 35, 130 ± 36.5, 767.8 ± 281.1, 910 ± 372.7, 33.9 ± 7.5, and 658.4 ± 164.6 (7α-, 16β-, 2α-, 16α-, 2β-, and 6β-hydroxy-testosterone, respectively); 4.86 ± 1.28 (7-hydroxy-warfarin); and 3.22 ± 0.34 (nmol/mg protein/min, 6-hydroxy-chlorzoxazone).

The activities of CYP2C6 (warfarin 7-hydroxylation) and CYP2A (testosterone 7α-hydroxylation) were not significantly affected by iloperidone (Figure 1).

### 2.2. The Influence of Two-Week Treatment with Iloperidone on the CYP Protein Level in Rat Liver Microsomes

**Figure 2:**
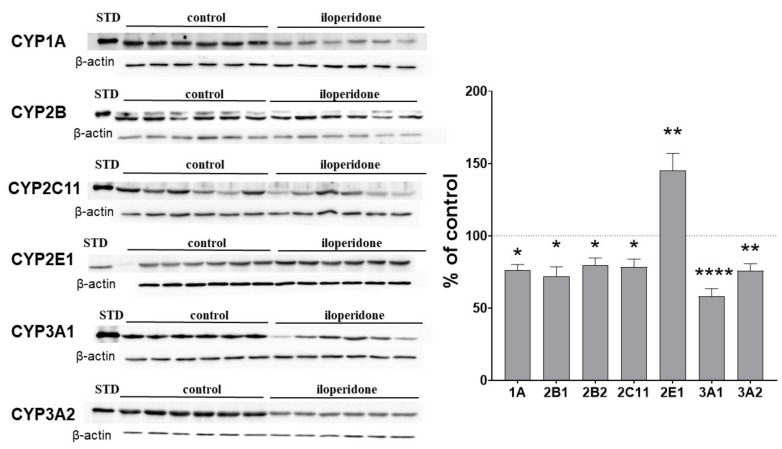
The effect of two-week treatment with iloperidone on the protein levels of CYP1A, CYP2B, CYP2C11, CYP3A, and CYP2E1 enzymes in rat liver microsomes. Microsomal proteins (10 μg) were subjected to the Western immunoblot analysis. The results are presented as representative blots from six (for the control and for iloperidone treatment) separate rats per treatment. The data are expressed as the mean ± S.E.M. (*n* = 12). Statistical significance was assessed by Student’s *t*-test and marked as * *p* < 0.05, ** *p* < 0.01, **** *p* < 0.0001, compared with the control.

CYP enzymes that are important for drug metabolism, and for which significant activity changes were detected, were then chosen for expression measurements at the mRNA and protein levels. The observed changes in CYP protein levels corresponded well with the alteration in CYP activities. The CYP1A protein level fell down to about 75% of the control. The levels of CYP2B1 and CYP2B2 proteins were reduced by the neuroleptic to 72% and 79% of the control, respectively. Iloperidone significantly decreased the CYP2C11 protein to 78% of the control. The CYP3A1 and CYP3A2 protein levels were lowered by iloperidone to 58% and 75% of the control. On the other hand, the neuroleptic increased the CYP2E1 protein level up to 145% of the control (Figure 2).

### 2.3. The Effect of Iloperidone Treatment on the mRNA Level of CYP Enzymes in the Liver

**Figure 3:**
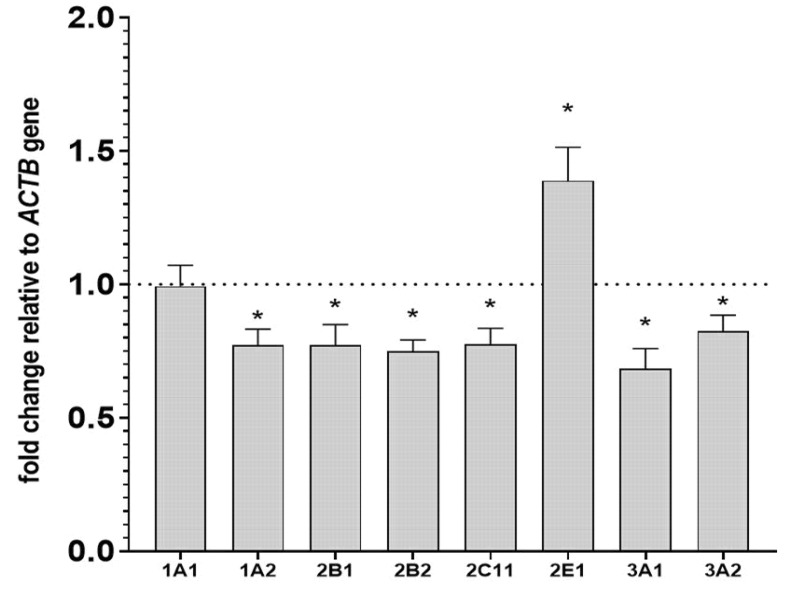
The effect of two-week treatment with iloperidone on the mRNA levels of *CYP1A*, *CYP2B*, *CYP2C11*, *CYP3A*, and *CYP2E1* genes in the liver. The results are expressed as the fold-change in relation to the *ACTB* housekeeping gene. All the values are the mean fold-change calculated by the comparative delta-delta Ct method for the control and iloperidone-treated rats. All values are the means ± S.E.M. (*n* = 10). The significance of differences between the results was calculated using Student’s *t*-test. Statistical significance is shown as * *p* < 0.05 vs. control group.

In parallel with the changes in activity and protein levels, the investigated neuroleptic produced a significant decrease in the mRNA level of the *CYP1A2* gene down to 77% of the control. Iloperidone reduced the *CYP2B1* and *CYP2B2* mRNA levels to 77% and 74% of the control. The level of *CYP2C11* mRNA was diminished to 77% of the control after iloperidone treatment. The *CYP3A1* and *CYP3A2* mRNA levels were reduced by the investigated drug to 68% and 82% of the control, respectively. The examined drug did not produce any significant changes in the *CYP1A1* mRNA levels. On the other hand, the neuroleptic increased the *CYP2E1* mRNA level to 138% of the control (Figure 3).

### 2.4. The Effect of Two-Week Treatment with Iloperidone on the Pituitary GHRH and Serum Concentrations of Hormones and Cytokines

**Figure 4:**
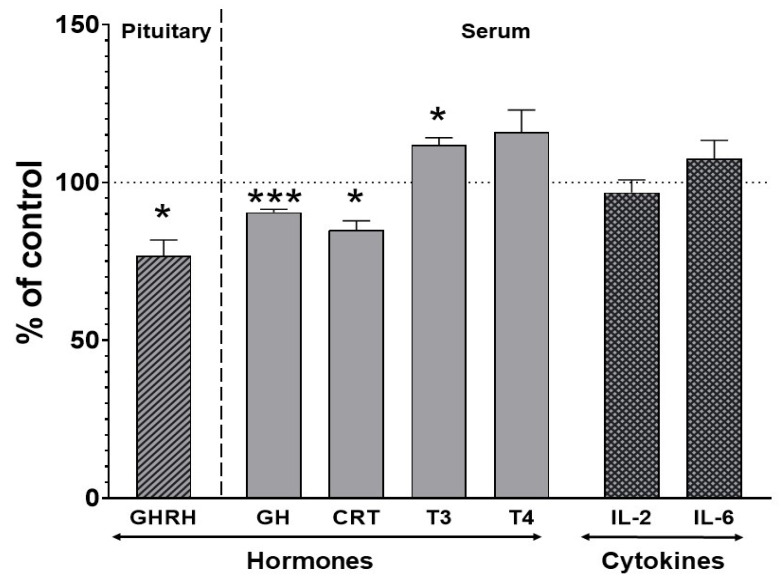
The effect of two-week treatment with iloperidone on the levels of pituitary and serum hormones and serum cytokine concentrations. All the values are the mean ± S.E.M. of the control (*n* = 12) and iloperidone-treated (*n* = 12) group. Statistical significance was assessed by Student’s *t*-test and is shown as * *p* < 0.05 or *** *p* < 0.001 compared with the control. The absolute control values were 34.14 ± 4.02 ng/mg for pituitary growth hormone-releasing hormone (GHRH) and 6.3 ± 0.16 ng/mL, 18.19 ± 2.91 ng/mL, 1.12 ± 0.054 ng/mL, 3.03 ± 1.18 ng/mL, 66.06 ± 8.39 ng/mL, and 1.1 ± 0.15 ng/mL for serum growth hormone (GH), corticosterone (CRT), triiodothyronine (T_3_), thyroxine (T_4_), interleukin-2 (IL-2), and interleukin-6 (IL-6), respectively.

The level of growth hormone-releasing hormone (GHRH) in the pituitary gland decreased to 76% of the control after chronic treatment with iloperidone. The ELISA test revealed a significant decrease in the serum concentration of corticosterone and growth hormone (to 84% and 90% of the control, respectively) and an increase in the thyroid hormone triiodothyronine (T_3_) (to 111% of the control). The concentration of thyroxine (T_4_) was not significantly changed by iloperidone treatment (Figure 4). No changes in the serum concentration of the investigated interleukins (IL-2 and IL-6) were observed after chronic iloperidone treatment (Figure 4).

## 3. Discussion

To achieve a desired therapeutic effect in schizophrenia patients and to maintain their mental wellbeing, pharmacological therapy needs to be continued for a long time, usually from the onset of symptoms and for the rest of the patient’s lives. Such a long therapy, which is often prescribed in combination with different drugs, may influence the expression of biologically active proteins including drug metabolizing enzymes and may lead to metabolic interactions. This work is the first report showing changes in the expression and activity of cytochrome P450, the main enzyme responsible for oxidative drug metabolism, which are produced by chronic treatment with pharmacological doses of the novel atypical neuroleptic iloperidone.

The results show a broad spectrum of changes in liver CYP enzymes, which are important for the metabolism of drugs (CYP1A, CYP2B, CYP2C, and CYP3A) and small toxic xenobiotics (CYP2E1). It is worth noting that the observed decreases in the CYP enzyme activities correlate well with simultaneous lowering in the enzyme protein and mRNA levels, which points to the inhibition of the transcription of genes coding for those CYP proteins (i.e., *CYP1A2*, *CYP2B1*, *CYP2B2*, *CYP2C11*, *CYP3A1*, and *CYP3A2*). Interestingly, the decreases in the expression and activity of the abovementioned CYP genes were accompanied with parallel changes in CYP-regulating hormone levels.

The diminished levels of pituitary growth hormone-releasing hormone (GHRH) and serum growth hormone (GH) correlated positively with the decreased expression of *CYP2C11* and *CYP3A1/2* in the rat livers. GH produced by the pituitary and acting via its hepatic membrane receptor (GHR) is the main positive regulator of *CYP2C11* expression and plays an important role in the upregulation of *CYP3A* genes [20,21]. The diminished concentration in serum corticosterone, a positive regulator of the *CYP1A*, *CYP2B*, and *CYP3A* genes acting via its nuclear receptor (GR) [22,23,24,25,26], also positively correlated with the observed reduction in the expression of those genes in the iloperidone-treated rats. On the other hand, an increase in the serum concentration of thyroid hormone T_3_, which is known to negatively influence the cytochrome P450 expression via its nuclear receptor (THR) [27,28,29], negatively correlated with the decreased expression of the CYP1A, CYP2B, CYP2C, and CYP3A enzymes found after prolonged administration of iloperidone. Our results are in line with the known molecular mechanisms of cytochrome P450 regulation by the cytosolic aryl hydrocarbon receptor (AHR) or the nuclear receptors: pregnane X receptor (PXR), constitutive androstane receptor (CAR), and retinoic X receptor (RXR). GR receptors contribute to the regulation of CYP3A, CYP2B, and CYP2C genes through a direct or an indirect molecular mechanism, including a functional cross-talk among GR, PXR, CAR, and RXR. The expressions of PXR, CAR, and RXR are activated by glucocorticoids and xenobiotics [25,30,31,32]. AhR, which is primarily responsible for the regulation of CYP1A enzymes, is also modulated by physiological levels of glucocorticoids and sex hormones [23]. Natural steroids, such as androgens or pregnanes, which are ligands for the nuclear receptors PXR and/or CAR, can directly affect CYP2B, CYP2C, or CYP3A expression in the liver [33]. Therefore, it seems that iloperidone indirectly affects cytochrome P450 via a decrease in corticosterone concentration and, in turn, decreased activation of GR, though a direct action on one of the abovementioned nuclear receptors cannot be excluded. It is worth noting that the serum concentrations of interleukins, which downregulate CYP enzymes (IL-2 and IL-6), were not changed by iloperidone. The activity of CYP2C6 enzyme, which is less vulnerable to hormonal changes, was not affected by iloperidone treatment.

In contrast with the above-discussed decreases in the expression and activity of the CYP1A, CYP2B, CYP2C11, and CYP3A enzymes, the expression and activity of the CYP2E1 enzyme was increased by chronic iloperidone treatment. The CYP2E1 enzyme is less engaged in drug metabolism compared with other members of CYP2 family and is involved only in the biotransformation of a few drugs (chlorzoxazone, acetaminophen, isoniazid, lidocaine, coumarin derivatives, and gaseous anesthetics). However, it is important for the metabolism of small-molecular hydrophobic compounds, such as acetone or alcohol, and catalyzes the oxidation of procarcinogens (vinyl chloride or bromide, dimethyl- or diethyl-nitrosamine, acrylonitrile, urethane, styrene, benzene, CCL4, chloroform, and trichloroethylene) [34]. Therefore, the induction of CYP2E1 is not desirable for maintaining the correct (safe) rate of biochemical processes in the organism [35,36]. Alcohol, acetone, and long starvation induce the enzyme, and iloperidone seems to have a similar property, though its effect on the enzyme activity is less potent. CYP2E1 is known to be induced at different levels, i.e., at the transcriptional, posttranscriptional, and posttranslational levels [37,38,39,40,41]. In the case of the investigated neuroleptic, regulation at the transcriptional level occurs, as the drug increases the *CYP2E1* mRNA, CYP2E1 protein, and enzyme activity with good positive correlation. The observed increase in CYP2E1 expression and activity may be connected (at least partially) with iloperidone-evoked inhibition of the GHRH-GH hormonal axis and increased T_3_ serum concentration. It has been demonstrated in different animal models that CYP2E1 is negatively regulated by GH [42,43,44,45,46,47,48] but positively by T_3_ [49]. Since the CYP2E1 enzyme is well conserved among species, its regulation may be similar in rats and humans. Thus, further studies in this direction concerning human CYP2E1 in relation to different iloperidone doses are advisable to find out whether the drug is capable of enhancing the enzyme activity in the human liver.

**Figure 5:**
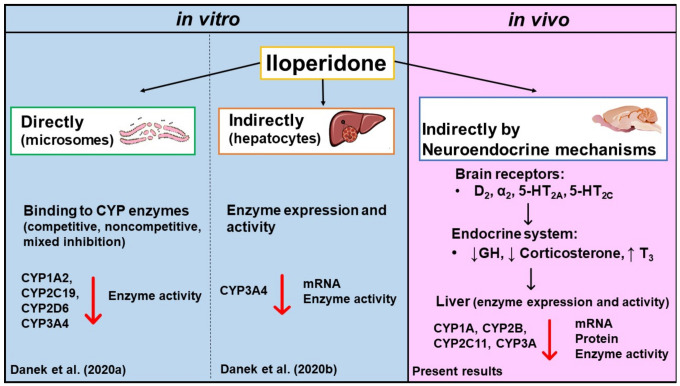
The effect of iloperidone on liver cytochrome P450 shown in different experimental models. The engagement of direct and indirect mechanisms in the interaction between the neuroleptic and CYP enzymes.

Considering our previous results obtained using in vitro models and the present results obtained in vivo after chronic treatment with iloperidone, it can be concluded that the neuroleptic can affect liver cytochrome P450 via different direct and indirect mechanisms operating at the level of the liver and the whole organism, which involve (1) direct inhibition by binding to the enzyme via mixed, competitive, or noncompetitive mechanism, as shown for CYP2C19, CYP2D6, and CYP3A4, respectively, in liver microsomes [18]; (2) reduction in enzyme expression and activity at the level of hepatocyte, as shown for CYP3A4 in cell culture [19]; and (3) neuroendocrine regulation, as shown in the present in vivo study for many CYP enzymes after chronic treatment with iloperidone (Figure 5).

The observed inhibitory effect of iloperidone on the CYP1A, CYP2B, CYP2C, CYP2D, and CYP3A subfamilies may lead to pharmacokinetic (metabolic) interactions with co-administered drugs. Considering the similarities in regulation, amino acid sequence homology, and function between the tested rat CYPs and respective human CYPs, pharmacokinetic interactions with CYP-metabolized substrates (steroids, drugs, and carcinogens) can be expected in iloperidone-treated patients. Thus, the presented results may have serious medical implications. Changes in patient susceptibility to drugs should be taken into account by physicians who also need to monitor pharmacotherapy. For example, newer antidepressants and second-generation antipsychotic drugs are often used by clinicians, which may lead to pharmacokinetic and pharmacodynamic interactions [50]. On the other hand, stimulation of the CYP2E1-mediated metabolism of drugs, alcohol, and procarcinogens by iloperidone should also be considered and further investigated.

## 4. Materials and Methods

### 4.1. Chemicals and Reagents

Iloperidone was obtained from TargetMol (Boston, MA, USA). Steraloids (Newport, KY, USA) provided testosterone and its metabolites. Caffeine and its metabolites, chlorzoxazone and its metabolite 6-hydroxychlorzoxazone, bufuralol and its metabolite 1′-hydroxybufuralol, glucose-6-phosphate-dehydrogenase, glucose-6-phosphate, NADP, NADPH, RNA-free water, and Tween 80 were purchased from Sigma (St. Louis, MO, USA). Warfarin was donated by Merck (Darmstadt, Germany). 7-Hydroxywarfarin was synthesized at the Maj Institute of Pharmacology, Kraków, Poland (Daniel et al. 2006). For RNA isolation, a Total RNA Mini kit purchased from A&A Biotechnology (Gdynia, Poland) was used. A High-Capacity cDNA Reverse Transcription Kit, TaqMan assays, and the TaqMan Gene Expression Master Mix were supplied by Life Technologies (Carlsbad, CA, USA). The primary rabbit polyclonal anti-rat CYP1A1/2, CYP3A1, and CYP3A2 antibodies (Millipore, Temecula, USA); anti-rat CYP2C11 and CYP2E1 antibodies (Thermo Fisher Scientific, Walthman, MA, USA); monoclonal mouse anti-rat CYP2B1/2B2 (Santa Cruz Biotechnology, Dallas, TX, USA); and polyclonal anti-rat β-actin antibody (Sigma, St. Louis, MO, USA) were used. Horseradish peroxidase-labeled secondary antibodies, goat anti-mouse antibodies (Jackson ImmunoResearch, West Grove, PA, USA), and goat anti-rabbit antibodies (Vector Laboratories, Burlingame, CA, USA) were used. Rat cDNA-expressed CYP1A2, CYP2B1, CYP2C11, CYP2E1, CYP3A1, and CYP3A2 (Supersomes) were from Gentest Corp. (Woburn, MA, USA). The chemiluminescence reagents SuperSignal West Pico PLUS Chemiluminescent Substrate kit came from Thermo Fisher Scientific (Walthman, MA, USA). The ELISA kits for growth hormone, corticosterone, T_3_, T_4_, Il-2, and Il-6 were obtained from Bioassay Technology Laboratory (Bioassay Technology Laboratory, Shanghai, China). The kit for growth hormone-releasing hormone (GHRH) came from MyBiosource (San Diego, CA, USA). All necessary chemicals of the highest purity used for analysis by high-performance liquid chromatography (HPLC) were donated by Merck (Darmstadt, Germany).

### 4.2. Animals

Male Wistar Han rats (Charles River Laboratories, Sulzfeld, Germany), three months old and weighing 280–300 g, were initially acclimatized and housed (6 per cage) in environmentally controlled rooms (ambient temperature 22 ± 2 °C, humidity 50 ± 5%, and 12:12 light:dark cycle). The animals had free access to tap water and typical laboratory food. All animal procedures were conducted in strict accordance with the European regulations for animal experimentation on the Protection of Animals Used for Scientific Purposes (EU Directive 2010/63/EU). The experimental protocols were approved by the Local Ethics Commission for Experimentation on Animals at the Maj Institute of Pharmacology, Polish Academy of Sciences, Kraków.

### 4.3. Drug Treatment and Liver Sample Preparation

The rats (*n* = 12 for each treatment group) received intraperitoneal injections of iloperidone (1 mg/kg) or vehicle control (1% Tween 80 in sterile water) once daily for a period of two weeks. The iloperidone solution for injection was prepared daily. The selected dose of iloperidone was active in pharmacological and behavioral paradigms [51,52,53]. The rats were decapitated 24 h after the last dose. The livers were quickly removed, frozen in dry ice, and stored at −80 °C. The blood was collected, and the serum was separated by centrifugation and stored at −80 °C. Liver microsomes were prepared from individual rats by differential centrifugation in 20 mM Tris/KCL buffer (pH 7.4), including washing with 0.15 mM KCL to remove the drug administered in vivo, according to the previously used method [54].

### 4.4. CYP Enzyme Activities in the Liver

The metabolism of caffeine, warfarin, chlorzoxazone, and testosterone was investigated in vitro using liver microsomes at a linear dependence of product formation on time, protein, and substrate concentration. The activity of CYP1A was determined by measuring the rate of caffeine metabolism (C-8-hydroxylation and 3-N-demethylation) at a substrate concentration of 100 μM and incubation time of 50 min. Caffeine and its metabolites were analyzed by HPLC with UV detection [55]. The activity of CYP2C6 was studied by measuring the rate of warfarin 7-hydroxylation at a substrate concentration of 60 μM and incubation time of 15 min. Warfarin and its metabolite were analyzed by HPLC with fluorescence detection [56]. The activity of CYP2E1 was estimated by measuring the rate of 6-hydroxylation of chlorzoxazone at a substance concentration of 200 μM and incubation time of 20 min. Chlorzoxazone and its metabolites were analyzed by HPLC with UV detection [17]. The activities of CYP2A, CYP2B, CYP2C11, and CYP3A were studied by measuring the rates of cytochrome P450 enzyme-specific reactions: 7α-, 16β-, 2α-, 16α-, 2β-, and 6β-hydroxylation of testosterone at a substrate concentration of 100 μM and incubation time of 15 min. Testosterone and its metabolites were analyzed by HPLC with UV detection [57,58].

### 4.5. Determination of CYP Proteins in Liver Microsomes

The protein levels of the examined CYP enzymes (CYP1A, CYP2B1, CYP2B2, CYP2C11, CYP2E1, CYP3A1, and CYP3A2) in the liver microsomes of control (*n* = 12) and iloperidone treated rats (*n* = 12) were estimated using Western immunoblot analyses. In brief, microsomal proteins (10 μg) were separated on 12% sodium dodecyl sulfate-polyacrylamide gels in a Laemmli buffer system and transferred onto a nitrocellulose membrane. The blots were probed with primary antibodies against appropriate CYP enzymes: monoclonal anti-rat CYP2B and polyclonal anti-rat CYP1A1/2, CYP2C11, CYP2E1, CYP3A1, and CYP3A2. Then, the blots were incubated with the appropriate (anti-mouse IgG or anti-rabbit IgG) horseradish peroxidase-conjugated secondary antibodies, and the bands were visualized by enhanced chemiluminescence. Rats that cDNA-expressed CYP1A2, CYP1B1, CYP2C11 (5 μg), CYP2E1 (2 μg), CYP3A1, CYP3A2 (1 μg) were used as respective standards. The immunoblots were evaluated using a luminescent image analyzer (LAS-1000, (Fuji Film, Tokyo, Japan), and the relative levels of immunoreactivity were quantified using the Image Gauge software (Fuji Film, Tokyo, Japan). The data were normalized to protein based on the β-actin levels.

### 4.6. Analysis of mRNA Level in the Liver

The total RNA was isolated from frozen liver tissue using a Total RNA Mini kit following the manufacturer’s instructions. The RNA was reverse transcribed using a High-Capacity cDNA Reverse Transcription Kit according to the manufacturer’s instructions. The expression of the genes encoding the cytochrome P450 enzymes *CYP1A1* (Rn01418021_g1), *CYP1A2* (Rn00561082_m1), *CYP2B1* (Rn01457880_m1), *CYP2B2* (Rn02786833_m1), *CYP2C11* (Rn01502203_m1), *CYP2E1* (Rn00580624_m1), *CYP3A1* (Rn03062228_m1), and *CYP3A2* (Rn00756461_m1) and the reference gene encoding beta-actin *ACTB* (Rn00667869_m1) were detected by a real-time polymerase chain reaction (PCR) using TaqMan Gene Expression Master Mix and species-specific TaqMan type probes and primers (TaqMan Gene Expression Assay, Life Technologies). Amplification was performed under the following conditions: 50 °C for 2 min and 95 °C for 10 min followed by 40 cycles at 95 °C for 15 s and 60 °C for 1 min. Real-time PCR runs were performed using the Bio-Rad CFX96 PCR system (Bio-Rad, Hercules, CA, USA). Gene expression was determined by the 2-delta Ct method using *ACTB* gene expression as a reference.

### 4.7. Analysis of Hormones and Cytokines in the Pituitary and Blood Serum

The levels of pituitary GHRH and serum concentrations of hormones (GH, corticosterone, T_3_, and T_4_) and cytokines (IL-2 and IL-6) of the control (*n* = 12) and chronically iloperidone-treated rats (*n* = 12) were measured using ELISA kits following the manufacturer’s instructions. The pituitaries were homogenized in phosphate-buffered saline (pH = 7.0; 1:20 *v*/*v*). The homogenates were frozen on dry ice three times and then centrifuged for 5 min at 5000× *g*. Absorbance was measured using a Synergy/HTX multi-mode reader (BioTek, Winooski, VT, USA).

### 4.8. Statistical Data Analysis

The statistical significance of changes in enzyme activity, protein level, gene expression, or hormones/cytokine levels was estimated using Student’s *t*-test (GraphPad Prism Software Inc., San Diego, CA, USA). The results were regarded as statically significant when *p* < 0.05. The obtained results are presented as the mean ± S.E.M.

## 5. Conclusions

In summary, chronic treatment with iloperidone produces broad changes in cytochrome P450 metabolizing enzymes and cytochrome P450-regulating hormone levels, which points to neuroendocrine regulation of the investigated CYP enzymes, resulting from the central receptor profile of the neuroleptic targeting monoaminergic systems of the brain. It has been documented that dopaminergic D_2_ [12], adrenergic α_2_ [13], and serotonergic 5-HT_1A_ and 5-HT_2C_ [15] receptors of the brain are engaged in the central neuroendocrine regulation of liver cytochrome P450. The action of iloperidone on dopaminergic, serotonergic, and adrenergic receptors may affect the neuroendocrine system and, in turn, the regulation of cytochrome P450 in the liver (discussed in [17]). The observed inhibitory effect of iloperidone on cytochrome P450 enzymes of the CYP1A, CYP2B, CYP2C, and CYP3A subfamilies may lead to pharmacokinetic (metabolic) interactions with concomitantly administered drugs. Moreover, the stimulation of CYP2E1-mediated metabolism of drugs, alcohol, and procarcinogens by iloperidone should also be considered and further investigated.

## Data Availability

The data are contained within the article.
